# A Focus on Aging, HIV/AIDS, and Neurocognitive Challenges: Examining Southern Nevada HIV Sector Providers’ Awareness and Prospective Roles

**DOI:** 10.3390/ijerph20196876

**Published:** 2023-10-02

**Authors:** Brandon Ranuschio, Sherry Bell, Jason D. Flatt, Lianne Barnes, Trinity Puno, Alexander Ribeiro, Nadia Sheik-Yosef, Esmeralda Villalobos, Janelle Wackens, Renato M. Liboro

**Affiliations:** 1 Department of Psychology, College of Liberal Arts, University of Nevada, Las Vegas, NV 89154, USA; fraga@unlv.nevada.edu (B.R.); bells12@unlv.nevada.edu (S.B.); lianne.barnes@unlv.edu (L.B.); puno@unlv.nevada.edu (T.P.); ribeia1@unlv.nevada.edu (A.R.); sheikn1@unlv.nevada.edu (N.S.-Y.); villae12@unlv.nevada.edu (E.V.); wackens@unlv.nevada.edu (J.W.); 2 Department of Social and Behavioral Health, School of Public Health, University of Nevada, Las Vegas, NV 89154, USA; jason.flatt@unlv.edu; 3 Centre for Addiction and Mental Health, Toronto, ON M5S 2S1, Canada

**Keywords:** aging, healthcare and service providers, HIV/AIDS, HIV-associated neurocognitive disorder, neurocognitive challenges, older people living with HIV/AIDS

## Abstract

Although abundant research has been carried out to investigate the underlying mechanisms that may cause neurocognitive challenges among middle-aged and older people living with HIV/AIDS (PLWH), to monitor the prevalence rates of HIV-related neurocognitive deficits, and to identify factors related to the improvement of diagnostic screening tools, classification and nosology, and clinical and rehabilitative treatment of HIV-Associated Neurocognitive Disorder (HAND); to date, there have been only a few studies that have explored and examined the awareness and work experiences HIV sector healthcare and service providers have related to HAND. To address this research gap, we conducted a qualitative, community-based participatory research study and interviewed 12 HIV sector providers in Southern Nevada, USA, from January to April 2022. After performing a thematic analysis of our interviews, we were able to identify two major themes and several sub-themes. Under our first major theme, provider awareness and knowledge, we identified four sub-themes: (1) prior knowledge and current awareness; (2) lived experiences of patients and clients with neurocognitive challenges; (3) lack of knowledge as a barrier to providing needed care; and (4) continuing education and professional development. Under our second major theme, prospective provider roles, we identified three sub-themes: (1) early detection; (2) direct and practical support; and (3) appropriate and timely referrals. In this article, we discuss our findings and lessons learned from our study, as well as their implications for the future work of researchers and providers in the HIV sector related to improving care and support for people aging with HIV/AIDS and experiencing neurocognitive challenges.

## 1. Introduction

As healthcare and service providers in the HIV sector across the globe have strived to improve the care and support they provide for people living with HIV/AIDS (PLWH) [1,2], great strides have been made in the treatment of HIV/AIDS since the beginning of the epidemic in the 1980s [1,3]. The introduction of combination antiretroviral therapy (cART) during the mid-1990s has had a profound effect on the advancement of different treatment options. Novel and effective viral suppression strategies using cART have been developed in recent decades, which have dramatically reduced the risk of PLWH developing AIDS-related conditions, decreased their HIV-associated mortality, improved their survival rates, and greatly increased their life expectancy [4,5,6,7,8]. Despite such treatment advancements with the use of cART, PLWH have markedly continued to experience neurocognitive challenges as they age [8,9]. In particular, HIV-Associated Neurocognitive Disorder (HAND) among PLWH, especially among middle-aged and older adults, has persisted as a public health concern in both developed and developing countries [4,6,7,8,10]. 

HAND refers to a spectrum of neurocognitive challenges that may be experienced by PLWH in the realms of attention, executive functioning, language, learning, memory, motor skills, and sensory perception [11,12,13]. Even with the effectiveness of current HIV treatments, the prevalence of HAND has remained high, with rates that range from 45% to 64%, based on different studies investigating distinct settings [5,8,12,14]. Neurocognitive challenges could be present even among PLWH with well-controlled viremia [10], as milder forms of HAND have become even more prevalent in recent years [13,15]. Mild to moderate forms of neurocognitive challenges have been reported to significantly impact the adherence to medications, instrumental activities of daily living, and overall quality of life of PLWH [4,8,16,17]. 

The exact mechanisms that lead to the development of HAND are still under research [18]. Since HAND is a heterogenous condition, PLWH experiencing a cluster of neurocognitive challenges may have very different disease processes as they age [19]. Research has suggested that the pathogenesis of HAND in the era of cART is multifactorial, with possible contributions from central nervous system damage that occurs before the initiation of cART, chronic immune activation, cART neurotoxicity, neurodegenerative diseases, premature aging, and age-related comorbidities such as cardiovascular and cerebrovascular diseases, diabetes, and hyperlipidemia [15]. Complex interactions associated with aging, HIV/AIDS, and neurocognitive challenges have been investigated, and it appears that PLWH develop age-related brain health issues sooner compared to their HIV-negative counterparts, despite achieving effective viral suppression with cART [5,15].

With life expectancy and the estimated prevalence rates of neurocognitive challenges among PLWH remaining consistent in the 21st century [5,8,12,14], it would be reasonable to anticipate that PLWH will need to access healthcare, social services, and support programs from the HIV sector that will help them address the impact and consequences of HAND on their day-to-day lives as they grow older [7]. The management of HAND will be increasingly important for PLWH, and for the healthcare and service providers who support them by meeting their needs and promoting their wellbeing [10]. 

Current best clinical practices recommend that healthcare providers working with PLWH should include routine screening for HAND and appropriate referrals to diagnosticians and HIV specialists in their growing list of responsibilities [10,13]. This recommendation has been based on the premise that early detection of HAND is important to facilitate the adequate treatment and management of HAND [10]. Earlier studies have documented that clinicians using global deficit scores have been able to accurately classify neurocognitive impairments among PLWH [20], and that there has been high interrater reliability among neuropsychologists independently rating the presence and severity of neuropsychological impairment of HIV-positive patients [21]. More recent studies have reported that healthcare providers in occupational treatment settings have started to receive training and resources to conduct neurocognitive screening among PLWH, and, in the not-so-distant future, could prospectively be able to perform this screening without prejudicing patients and clients in the workplace [10]. It remains to be seen if healthcare providers in settings other than occupational health, and service providers with little to no clinical background, will subsequently receive such training and resources to conduct neurocognitive screening. Related to this, it is not clear at this time how familiar or knowledgeable most healthcare and service providers are of HAND and the persistent public health concern that is associated with it [6,7,10].

Although abundant research has been conducted to investigate the underlying mechanisms that lead to the neurocognitive challenges of PLWH [5,15,19,22]; to monitor the prevalence rates of HIV-related neurocognitive deficits [13,17,22,23,24]; and to identify factors related to the improvement of diagnostic screening tools [13,15,22,25,26], classification and nosology [8,27,28], and clinical and rehabilitative treatment of HAND [4,22]; to date, there have been only a few studies that have explored and examined the awareness and work experiences healthcare and service providers working with PLWH have related to HAND [6,7,10]. For instance, a recent study that was conducted by Gouse and colleagues revealed that 80% of HIV sector providers in South Africa had heard of HAND before participating in their study, but only 11% have screened for it in their work, and only 2% have received appropriate training to address it [10]. The study reported that lack of expertise on HAND was the largest obstacle providers experienced to confidently address it, and that 77% of providers thought it would be important to screen for HAND, which suggested that they found value in gaining knowledge about HAND but have not had the opportunity to learn more about it [10]. In a qualitative study that was conducted in Southwestern and Central Ontario, Canada, research findings revealed that service providers in the HIV sector faced numerous personal and professional barriers (e.g., limited knowledge about HAND), service access barriers (e.g., limited access to needed services with adequate expertise and experience working with PLWH), and systemic barriers (e.g., lack of funding in the HIV sector) while addressing HAND in their work [7]. These barriers prompted service providers to develop and employ intrapersonal strategies (e.g., staying informed about HAND using online sources), interpersonal strategies (e.g., providing practical assistance for the memory-impaired), and organizational strategies (e.g., creating dedicated support groups) to help their clients who experienced neurocognitive impairment [6]. Further research on this focus still needs to be done to learn more about awareness, and the potential roles healthcare and service providers could assume to support PLWH experiencing neurocognitive challenges that could be attributable to HAND. 

While it might be plausible to assume that primary care providers (i.e., doctors, nurse practitioners, physician assistants) with a clinical practice focused on HIV/AIDS, psychiatrists, healthcare providers in occupational treatment settings, neuropsychologists, and HIV specialists and researchers would have a strong grasp of the underpinnings of HAND as a relevant condition that may significantly impact the lives of aging PLWH, it would be imprudent to surmise the awareness and work experiences other healthcare and service providers working with PLWH (specifically those in community-based settings) might have related to HAND without conducting systematic, empirical research. 

Based on this premise, we conducted a qualitative study in 2022 in Las Vegas, Nevada, USA, in order to gain a greater understanding of the awareness of community-based HIV sector providers regarding HIV-related neurocognitive challenges in our local setting. It is important to note that in 2020, there were 10,459 PLWH in Las Vegas, and that our city had an HIV prevalence rate of 655 per 100,000 people [29]. In the same year, the number of new HIV diagnoses in Las Vegas was 312, and 83% of all people diagnosed with HIV were documented to have a linkage to care, which meant that they had visited an HIV healthcare provider within one month of being diagnosed with HIV [29]. 

The primary research aims of our qualitative study described in this article are to (1) examine HIV sector community-based healthcare and service providers’ awareness of HAND, and (2) explore what providers are or may be able to do in their work to support PLWH experiencing neurocognitive challenges, particularly in the context of the HIV sector in Las Vegas and the surrounding region of Southern Nevada, USA. 

## 2. Materials and Methods

### 2.1. Partnerships and Collaborations

In close collaboration with our primary community partner, The LGBTQIA+ Community Center of Southern Nevada (The Center), we conducted the present qualitative study as part of a larger sequential mixed-methods [30] project utilizing a community-based participatory research (CBPR) approach [31,32,33] that principally sought to examine awareness of HAND among relevant stakeholders (i.e., PLWH and community-based healthcare and service providers) from Southern Nevada, and to explore their perspectives and lived or work experiences related to addressing neurocognitive challenges. For our focus on healthcare and service providers dedicated to serving PLWH, the Center was instrumental in linking us with a wide network of clinics, community health centers, community-based not-for-profit agencies, AIDS service organizations, and other relevant stakeholder groups from Southern Nevada, which were all dedicated to providing health and social services specifically to aging PLWH in our region. Our community partners actively participated in many phases of our project. They collaborated with us in determining our research focus, choosing our research method, recruiting our study participants, assessing and interpreting our findings from our data analysis, and disseminating our study findings and lessons learned to the rest of the Southern Nevada community. For this article, we discuss only our study findings and lessons learned based on the perspectives and work experiences of HIV sector providers from Southern Nevada. Our study findings and lessons learned based on the perspectives and lived experiences of middle-aged and older PLWH from Southern Nevada are beyond the scope of this article, and will be discussed elsewhere in another research article.

### 2.2. Participants

Before we began conducting our study, we sought and received ethics approval for our research protocol from the Institutional Review Board (IRB) of the University of Nevada, Las Vegas (IRB protocol # 1657448-4—9 August 2021). In order to recruit participants, we utilized printed flyers that we posted on the premises of our community partner organizations, agencies, and clinics, and recruitment messages that we made available through our community partners’ different email websites and listservs. We actively participated in numerous community events that our community partners organized and sponsored, which allowed us to directly hand out recruitment pamphlets and flyers to prospective participants during these events. Together with our community collaborators, we implemented a purposive sampling technique [34] to recruit our participants. Interested stakeholders were able to participate in our interviews if they met our inclusion criteria, which required them to be (1) 18 years of age or older at the time of the study, and (2) working as a community-based healthcare or service provider in the HIV sector of Southern Nevada for at least 6 months. We purposively selected providers interested in participating in our interviews who were not primary care providers (i.e., doctors, nurse practitioners, physician assistants) with a clinical practice focused on HIV/AIDS, psychiatrists, healthcare providers in occupational treatment settings, neuropsychologists, and HIV specialists and researchers since these providers were much more likely to stay current on the academic literature on HAND as a consequence of the expectations of their professions or jobs. We continued to recruit and interview participants until data saturation for key themes was achieved (i.e., no new information relevant to the key themes emerged as additional interviews were conducted). 

All of the 12 providers who participated in our interviews had direct and regular contact with middle-aged and older PLWH who have experienced neurocognitive challenges at some point in their lifetime since their HIV diagnosis. Our participants were counselors, community health workers, outreach workers, patient advocates, linkage-to-care coordinators, and case managers in the HIV sector of Southern Nevada. Their ages ranged from 33 to 64 years old, with a mean age of 47 and a standard deviation of 10.19. Half of our participants (n = 6) identified as female, and the other half (n = 6) identified as male. Half of our participants (n = 6) identified as straight, and the other half (n = 6) identified as gay. All of our participants identified as cisgender, and none of our participants identified as lesbian, bisexual, queer, transgender, or nonbinary in our sociodemographic questionnaire, which also provided them space and an opportunity to self-identify in the way they were most comfortable with or preferred. In terms of race, our participants identified as Black (50%, n = 6), White (40%, n = 5), and Asian-Pacific Islander (10%, n = 1). In terms of ethnicity, three of our participants (25%) identified as Hispanic, while the rest identified as non-Hispanic. At the start of their interview, we assigned each participant a pseudonym, and subsequently used their respective pseudonyms to identify them in this article. Each participant received a $50 gift card at the end of their interview as compensation for their efforts and the time they spent on the study.

### 2.3. Procedures and Material

We conducted our confidential semi-structured interviews with our 12 participants virtually over Zoom between January and April 2022, with each interview taking approximately one hour to complete. We developed and set our interview questions so that they would be open-ended, to allow participants to elaborate more freely with their responses. Our interview guide questions were focused on topics that we identified as important based on the survey findings of the recent quantitative study we conducted in Southern Nevada as part of our larger mixed-methods CBPR project that examined HIV sector providers’ awareness of HAND [30,35]. These questions were ratified by our community partners, and included topics such as work encounters with patients and clients, providers’ awareness of HAND, barriers to gaining knowledge on HAND or providing care to PLWH, and community resources to help address issues related to neurocognitive challenges experienced by PLWH [30,35]. Our interview guide included general questions to encourage interview participants to open up about their day-to-day work experiences such as, “Could you please tell me about your practice/work as a healthcare or service provider in the HIV sector of Southern Nevada?” It also included more probing questions that helped us learn more about their awareness of HAND (e.g., “How long has it been since you have been aware of the existence of HAND as a condition that affects PLWH?”), or any strategies or community resources that they were familiar with that may help support PLWH experiencing neurocognitive challenges (e.g., “What kind of strategies do you use, if any, to support your patients/clients living with HIV experiencing neurocognitive challenges?”) (Please see Table 1 for Interview Guide Questions).

In order to obtain constructive input from our community partners, we shared our survey results with them prior to creating a community report for distribution to the larger Southern Nevada community, and then we asked for their input. Not only did our community partners find our survey results noteworthy, but they also deemed it imperative for our research partnership to further explore the possible foundations and implications of our quantitative findings through one-on-one interviews with our survey participants who expressed interest in joining the qualitative stage of our CBPR project. Our research team’s research project coordinator conducted and digitally recorded our virtual interviews after receiving each of our participant’s express consent. We anticipated and addressed possible interview bias by having our interview guide questions reviewed and ratified by our community partners from the HIV sector prior to the conduct of our interviews. The interviews were then de-identified and transcribed by our research project coordinator and three other members of our research team using a structured transcription protocol. Transcriptions of the interviews that were transcribed by other members of our research team were meticulously cross-checked by our research project coordinator for accuracy prior to analysis. 

### 2.4. Analysis of Data

We analyzed our de-identified transcripts using the thematic analysis phases that have been established and recommended by Braun and Clarke [36]. Due to its inherent flexibility, we chose Braun and Clarke’s thematic analysis as our guiding framework to analyze our interview data. We deemed it the best approach to fulfill our study’s goals because its epistemological and theoretical freedom allowed for a flexible examination of the different perspectives we derived from our participants [36]. Braun and Clarke’s thematic analysis method is an iterative process that consists of six phases: (1) becoming familiar with the data, (2) generating codes, (3) generating themes, (4) reviewing themes, (5) defining and naming themes, and (6) locating exemplars for writing up [36]. To execute the first phase of our analysis, we used the first half of our data set of 12 interviews to establish an initial thematic codebook. Reviewing the first six transcripts of our study’s data set provided the more seasoned coders of our research team a considerable opportunity to familiarize themselves with our interview data. After perusing the first six participant interviews twice to become intimately familiar with the raw data, our research project coordinator and two of this article’s senior authors held face-to-face meetings as preliminary coders to discuss and agree on the possible major themes and sub-themes to include in the initial codebook. Our preliminary coders discussed at length which among our initial themes stood out as major themes that could serve as central organizing concepts for our codebook, and which initial themes could be subsumed under these major themes as sub-themes with distinct elements that had a natural fit under one of the major theme’s central organizing concepts. Upon reaching a consensus based on the review and initial coding of the first six interviews, our preliminary coders shared the contents of the initial codebook with the remaining members of our research team, who subsequently assisted in coding the remaining six interview transcripts to execute the second to fifth phases of our thematic analysis [36]. For the final phase of our analysis, members of our research team reviewed the remaining six interview transcripts as separate coders using the initial codebook as a guide, and then gathered together in regular, bi-weekly meetings to discuss their identified codes, and to finalize the themes and sub-themes from the interviews. Our research team uncovered 42 codes from our analysis, and we used an average of five to seven codes that eventually led to the generation of each of our themes and sub-themes. 

## 3. Results

We identified two major themes from our interview data. The two major themes we identified were: (1) provider awareness and knowledge, and (2) prospective provider roles. For each of these major themes, we were also able to identify a number of sub-themes, which we describe in detail below. 

### 3.1. Provider Awareness and Knowledge

It was evident in our interviews that all our participants recognized the importance of being aware that their patients or clients living with HIV/AIDS could potentially be or already have been experiencing neurocognitive signs and symptoms due to HAND. As they discussed the importance of being aware of the existence of HAND as a condition that could considerably affect the day-to-day functioning and quality of life of the PLWH they were working with in their jobs, several sub-themes became apparent in our analysis of our interview data. Under the first major theme, provider awareness and knowledge, we identified four sub-themes: (1) prior knowledge and current awareness; (2) lived experiences of patients and clients with neurocognitive challenges; (3) lack of knowledge as a barrier to providing needed care; and (4) continuing education and professional development. In the next sub-sections, we discuss these sub-themes and provide representative quotes to help elucidate them. 

#### 3.1.1. Prior Knowledge and Current Awareness

Most of our participants had some prior working knowledge of brain health as it relates to HIV/AIDS. The vast majority of our participants had already heard of PLWH developing HIV-associated dementia (HAD), which is very common knowledge among providers who had been working in the HIV sector for several years. Since nearly all of our participants (save for two) had already been working as HIV sector providers for four years or more, most of them had also already encountered at least one patient or client of theirs who had suffered from HAD. This prior knowledge of the existence of HAD as a condition that could affect PLWH made them aware that it was possible for any of their aging patients or clients to have neurocognitive challenges related to HIV/AIDS. Marianne (a straight, white female who had been working in the HIV sector for over five years) explained that she had a working knowledge of HAD based on her work experiences:

“Well, I know there’s such a thing as HIV dementia. I’ve had a number of older clients who I know would fall into that category [based on what I’ve learned in the past from reading about it]. You know, most of them are without a diagnosis, so it’s very difficult to say for sure. I have a number of clients who appear to have issues related to memory, and just cognition, in general.”

Although having prior knowledge of HAD had made most of them aware of the possibility that some of their patients and clients could develop or already had neurocognitive challenges, it was evident based on our interviews that many of our participants were not particularly aware that dementia was only at the severe end of the spectrum of neurocognitive challenges older PLWH could experience, and that for the most part, the more common manifestations of HAND among PLWH were mild to moderate since the advent of cART. Interestingly, some participants revealed that they had subsequently made efforts to learn more about HIV-related neurocognitive challenges by building on the knowledge they had about dementia that developed among PLWH. 

A few of our participants readily acknowledged that their participation in the survey we conducted for the quantitative stage of our larger mixed-method CBPR project improved their awareness and knowledge of HAND. Bob (a gay, black male who had been working in the HIV sector for over 10 years) shared, “Ever since the [HIV/AIDS] epidemic began, there had always been discussion of ‘HIV-related dementia’, is what I believe they called it. However, the specific designation ‘HAND’…it’s the first time I am actually learning of it in this study”. 

#### 3.1.2. Lived Experiences of Patients and Clients with Neurocognitive Challenges

Aside from gaining more awareness of different HIV-related neurocognitive challenges from building on their prior knowledge related to HAD, a majority of our participants were able to recall specific times when they encountered in their work PLWH who presented signs and symptoms that could have been attributable to HAND. While it takes more than one or two symptoms to even suspect that PLWH may have HAND, it was notable in the interviews that our participants were already attending to the neurocognitive decline of some of their patients and clients aging with HIV/AIDS. The lived experiences of their patients and clients experiencing neurocognitive challenges became an important catalyst for raising their awareness and knowledge of HAND. Franc (a gay, Asian-Pacific Islander male who had been working in the HIV sector for over one year) described the deterioration of some of his clients’ abilities to maintain their attention during their appointments:

“I’ll literally be working with someone, and I had to help them understand that, yes, they got their HIV care, but I’m like, oh my goodness, they’re not even paying attention to their care provider anymore. Their brain and their focus was literally withering away. I was re-explaining to them, ‘You’re going to a doctor for your HIV care, but they’re not your neurologist or psychiatrist.’ They were having difficulty paying attention and understanding that they needed to see another healthcare provider for their brain health issues.”

Beyond observing attention deficits, our participants also noticed memory-related challenges among some of their patients and clients, such as difficulties in making and keeping appointments or recalling recommended solutions to personal issues, in addition to their general forgetfulness. Marianne recalled a couple of her clients calling multiple times a day to repeatedly ask about the same concerns that they’d already began to resolve:

“I have two clients right now that called me five times already today. So they have definite memory issues and are just struggling to put the pieces together. We have one client who has a problematic situation, and they’re contacting different case managers across the city. I realize that they forget that they’ve already sought help and received it. What ends up happening, stuff gets complicated because they keep, you know, going in circles asking more and more people for help, and we don’t know that they’ve already asked for help from other people. So I give them advice to resolve their situation, not knowing somebody else is giving them a different piece of advice, and then they get confused and think that people are trying to trick them.”

Having worked for a very long time supporting PLWH, Steve (a, gay, white male who had been working in the HIV sector for over 20 years) reported that he was able to note who among his long-term clients began to display obvious signs of forgetfulness: “Among my clients, I could see the differences between those who had been taking their medications regularly and who had not. Some who had not been able to adhere to their treatment regimen eventually showed signs of early memory loss”.

Neurocognitive challenges can lead to significant difficulties in navigating the HIV-care continuum that PLWH rely upon on a regular basis to manage their health issues and related options. Accessing a community organization’s services often requires a large quantity of paperwork, which can be complicated as it is, and then can be exacerbated by a patient’s or client’s neurocognitive challenges. Patrice (a straight, black female who had been working in the HIV sector for over 10 years) shared how she supported a client with overcoming their issues related to the neurocognitive challenges they experienced by helping them access important resources:

“I had to basically walk them through in detail the paperwork and different steps needed so that they could access their social security benefits. I was able to experience that on a one-on-one basis, and I think they had like an education level of 3rd grade to begin with. Over time, I was able to see their cognitive function decline as they kept going on without getting medication.”

#### 3.1.3. Lack of Knowledge as a Barrier to Providing Needed Care

The vast majority of the providers we interviewed expressed in some way that a lack of knowledge on HAND poses a major barrier to accessing or providing care. This lack of knowledge can exist among both patients or clients and their providers. Both patients or clients and their providers need to have some awareness and knowledge of HAND in order to notice or look for the signs and symptoms of HAND. Gail (a straight, black female who had been working in the HIV sector for over five years) commented, “The biggest barrier is our lack of knowledge. If you don’t know [HAND] exists…you don’t know who to refer them to for their problems, and it’s hard to give them the help they need”. Gail continued to explain:

“I think, the biggest thing is just getting the information out there. That this is a problem that PLWH are facing, and could later be facing. I think a lot of the clients that I meet [minimize it and] attribute their challenges to age, and have made flippant comments like, ‘Oh, at this age, I have to write everything down’… not knowing that it could be an effect of living with HIV for so long.”

According to the participants, the main challenge to raising awareness and spreading knowledge is having a consistent source of reliable information. Participants noted that if HAND is not brought up or discussed in meetings, training, or seminars, then providers and the PLWH they work with would not even learn of its existence. Gail pointed out that providers’ knowledge could easily translate into knowledge useful to their patients or clients because of their regular provider and patient or client interaction:

“If they’re not aware, not educated, and if it’s not talked about, this can lead to their deteriorating health mentally and physically. It’s basically a lot of lack of knowledge, and it’s not discussed, you know. We need more provider education so that we could offer more education to clients.”

Our participants believed that raising awareness and sustaining knowledge are the first steps to enhancing early detection of and improving care for neurocognitive challenges. Victor (a gay, black male who had been working in the HIV sector for over three years) remarked that knowledge of the neurocognitive challenges that may develop or may already be present among people aging with HIV/AIDS should ideally be shared to frontline providers like them who are not necessarily specialists in HIV care but are responsible for supporting PLWH who may have substantial issues related to neurocognitive challenges. Based on Victor’s remarks, although HIV specialists and researchers who are entrenched in academic literature are likely already well aware of HAND, other providers who may engage with or provide services to middle-aged and older PLWH on a more regular basis should also be cognizant of the fact that their patients and clients could develop or already have HIV-related neurocognitive challenges. This is so that they can stay alert and keep an eye on the cognitive health of their patients and clients aging with HIV/AIDS. According to Victor:

“[Even] a primary care doctor [who does not specialize in HIV/AIDS] may not even recognize that certain symptoms are related to their patient’s HIV/AIDS because they haven’t necessarily been educated [about HAND]. ‘Cause I don’t think there’s a lot of people out there that know that part. So it’s the education of professionals and providers more than, you know, the people that are in the HIV community.”

#### 3.1.4. Continuing Education and Professional Development

In addition to bringing up the topic of their patients’ and clients’ brain health and neurocognitive challenges during meetings as necessary, and discussing them intermittently in once- or twice-a-year training or seminars, many participants believed that their need for current and updated information on HAND should be considered as an indication that they should have a more regular forum for continuing education and professional development activities on the relationships involving aging, HIV/AIDS, and neurocognitive challenges. In terms of information on HAND that is pertinent to providers, a few participants who had previous knowledge of it learned about it from routine continuing education opportunities. Marianne had some baseline knowledge of HAND, and explained that she first heard of it in seminars that she attended on a regular basis:

“Well, um, in the job that I do, I am constantly training. There’s constant training and continuing education. So I’ve attended hundreds of seminars, and that’s often brought up this topic. I think all providers should have regular training so they are able to help their clients better.”

Likewise, Patrice revealed that she gained her knowledge on HAND from taking part in regular training opportunities: “Over the years, I’ve attended trainings with the health department and Pacific AIDS Educations Training Center, where I learned about HIV-related neurocognitive challenges and HAND. I believe it’s important for providers to stay current with all kinds of information”. Even participants who had limited knowledge of HAND expressed their desire to attend future training and seminars that would cover the neurocognitive challenges related to HIV/AIDS. They recognized the importance of improving their understanding of HAND, and revealed that most of the information related to HIV/AIDS being taught in continuing education and the professional development opportunities they were able to attend tended to focus on medications to manage the virus and updates on cART. Gail elaborated:

“A lot of the ongoing information that we received is about, you know, new medications or new forms of treatment delivery, which we get from the actual drug companies themselves. They would send representatives to talk about their drugs. Getting some sort of resource on brain health would be really beneficial for us. Not only how to watch out for symptoms or perhaps even how to identify HAND, but also how to best address its impacts and what practical resources are out there for our clients to utilize.”

Having an acute awareness of the signs and symptoms to watch out for in their patients and clients, knowledge of what is already definitely known and documented by researchers and scientists about HAND, and vital information on the kind of strategies and resources that their aging patients and clients experiencing neurocognitive challenges could utilize, were some of the areas of knowledge that our participants wanted to deepen from attending continuing education and professional development opportunities.

### 3.2. Prospective Provider Roles

The second main theme that we identified from our interview data revolved around prospective provider roles that our participants and their colleagues could potentially play in the future, particularly in addressing the neurocognitive challenges of their patients and clients, as well as the adverse impacts of those challenges on their patients’ and clients’ medication adherence, activities of daily living, and overall quality of life. Based on their perspectives and work experiences, our participants had several ideas as to how they could be very helpful in terms of supporting PLWH experiencing neurocognitive challenges, which we categorized into three sub-themes. Under this second major theme, prospective provider roles, we identified three sub-themes: (1) early detection; (2) direct and practical support; and (3) appropriate and timely referrals. In the next sub-sections, we discuss these sub-themes and provide representative quotes to help elucidate them. 

#### 3.2.1. Early Detection 

Most participants readily pointed out the fact that, although screening for and diagnosing HAND were not part of their job descriptions, list of responsibilities, and skills set, they believed they could still play crucial roles in addressing the neurocognitive challenges of their patients and clients. As many of them met with their patients and clients often and/or on a regular basis, our participants thought that they could potentially play an important role in the early detection of their patients’ and clients’ neurocognitive challenges. If they had enough awareness and knowledge from training and other professional development activities that would give them enough confidence to note or recognize any changes in the neurocognition of the aging PLWH they worked with, especially over time, then they would be able to initiate a plan that could possibly prevent their patients’ and clients’ challenges from getting worse, as well as addressing some of the impacts of these neurocognitive challenges on their patients’ and clients’ daily lives. Robyn (a straight, black female who had been working in the HIV sector for over 10 years) remarked:

“I’m not a medical doctor, so I always try to encourage clients to talk to their physicians or see a health specialist within our community, especially when I notice something odd or different with the clients. The challenge at times, is a client has to acknowledge that there’s something going on. You know, some people, especially if they’re estranged from their families or friends, and they’re alone, it’s challenging for them to admit that they may be losing their independence or may require [professional] help from someone. So sometimes, it’s down to us to find creative ways to get them more help.”

Marianne agreed with these sentiments: “I suspect that anybody having neurocognitive challenges may soon experience progressive deterioration. Since we see our clients on a regular basis, we could help with detecting subtle changes early on so that we could support them sooner rather than later”. 

Another idea that was expressed by our participants involved assuming the role of patient advocate in light of the importance of the early detection of neurocognitive challenges. Although many providers may not be able to physically accompany patients and clients to seek help as they are already overwhelmed with numerous responsibilities, there are some providers who have it in their job description to personally advocate for the needs of the most vulnerable PLWH who seek their help. Janice (a straight, white female who had been working in the HIV sector for over one year) suggested:

“For example, at a doctor’s appointment for someone [with neurocognitive challenges] newly diagnosed or maybe trying to get back in care, there could be a case manager, patient advocate, or some provider who can help with bringing the client to someone who screens for different cognitive disorders. That person could be there with them, at least until a care plan is set in place.”

#### 3.2.2. Direct and Practical Support

In addition to the crucial role they could play in the early detection of their patients’ and clients’ neurocognitive challenges, our participants believed that they could be of great help in terms of supporting PLWH experiencing neurocognitive challenges by assisting them with managing the adverse impact of these neurocognitive challenges on their patients’ and clients’ daily lives. Although they recognized that it is beyond their capacity to initiate therapies that would treat the actual neurocognitive challenges their patients and clients are experiencing, many of our participants were confident that they would be able to assist the PLWH they worked with through the programs and services that are offered by the clinics, community health centers, not-for-profit agencies, and AIDS service organizations they worked in Southern Nevada. Our providers acknowledged that they could share with their patients and clients access to a wide array of programs and services that would help to address many of the adverse impacts of the neurocognitive challenges the PLWH were experiencing. The adverse impacts of the neurocognitive challenges that PLWH experience often create more difficulties in accessing their most basic needs, because the deficits PLWH develop most often relate to attention, decision-making, language, learning, memory, and problem-solving. Our participants emphasized that these basic needs are critical to the survival of PLWH but are mostly covered by the programs and services of the agencies and organizations that they work for in the community. Gail recounted the different programs and services, which her agency had been able to offer to her clients:

“A lot of it is food assistance, housing assistance, and emergency financial assistance for things like bills and utilities. Some are mental health programs or educational programs, things like lunch and learns, and group education events. With some patience and compassion from us, our programs and services could help our clients cope with concerns and problems they may have resulting from their brain health issues.”

The variety of programs and services in their workplaces was perceived as something very positive by our participants, who shared their passion for their work in their interviews. For instance, our participants who were case managers revealed that they would take on not just their clients’ HIV care, but also their various needs, aiding them by addressing other hardships that were linked to their neurocognitive challenges. Victor described the different hats that he wears at his job working through various workshops and housing programs, and providing education to clients:

“I am in charge of health education, the risk reduction to our clients who are virally suppressed, as well as creating new and exciting workshops that combat negative emotions and provide psychosocial support. Through these different programs, we’re able to help older folks with cognitive problems.”

There was a notable joy and pride in the voices of our participants as they shared in their interviews how they believe their work made a difference to PLWH struggling with different neurocognitive challenges. Many of our participants revealed that they chose to work in their roles in healthcare and support services, specifically to help PLWH, because they find great value in the work they do. Patrice shared with us that her work in case management keeps the job active, fresh, and exciting: “That’s why I love doing case management, and I stay in weekly meetings that list new resources. It includes housing, child care, clothing, jobs, social security, and more information from other case management providers”.

#### 3.2.3. Appropriate and Timely Referrals

Apart from potentially maximizing their role in providing direct and practical support for their patients and clients in the future, many of our participants also saw the crucial role they played and could continue to play in linking PLWH experiencing neurocognitive challenges to specific resources that their own clinics, organizations, and agencies may not offer, but that are offered by partner clinics, organizations, and agencies in the community. Our participants reported that the HIV sector network of providers in Southern Nevada is strong, tightly knit, and well connected. So another role they could play that would be critical to supporting PLWH experiencing neurocognitive challenges is helping assess patients’ and clients’ needs, and then providing them appropriate and timely referrals to other healthcare and service providers in their network. To support the varied needs of PLWH in Southern Nevada, providers reported that they have utilized their network of care that runs and relies heavily on referrals. Janice revealed that they have connected their clients to many healthcare practitioners across the region, “We have a long list of doctors we refer our clients to for all sorts of medical problems, including cognitive issues. We refer clients to family physicians, infectious disease specialists, neurologists, and psychiatrists”. Our participants, several of whom are case managers and linkage-to-care coordinators whose jobs involve fostering connections between clients and community organizations or not-for-profit agencies, have recognized the breadth of support some older PLWH they have been working with need.

According to our participants, providers in Southern Nevada have established a referral network in which they can get their patients and clients connected to almost any program or service PLWH may need. Clinics, community health centers, community-based not-for-profit agencies, and AIDS service organizations have been interconnected in their network in such a way that each of their unique contributions to the Southern Nevada HIV continuum of care combine to create a nearly comprehensive whole. Victor explained that the organization he works for is explicitly a referral organization wherein they have established and maintained multiple options to refer clients to address any need that comes up, “We’re only a referral based organization. We try to have a rolodex of as many providers and community partners as possible to help us. Perhaps the next step is to determine the hidden needs of PLWH with cognitive deficits”. These needs may involve aspects such as screening, patient intake, initiation of new cART treatments, and retention in care, in addition to the more practical supports that many providers offer in their places of work.

## 4. Discussion

As we examined HIV sector community-based healthcare and service providers’ awareness of HAND and explored what they are or may be able to do in their work to support PLWH experiencing neurocognitive challenges, we were able to obtain and analyze invaluable information based on our participants’ perspectives and work experiences. In terms of their awareness and knowledge of HIV-related neurocognitive challenges, providers seemed mostly to be aware of some of the brain health impacts of living with HIV/AIDS, but only a few were actually very familiar with HAND. Their awareness was either based on their prior knowledge of the possibility that dementia could develop among PLWH with poor access and/or adherence to cART and poor viral suppression, or on their work encounters with middle-aged and older patients and clients who exhibited neurocognitive deficits over time. Some participants revealed that they only became aware of HAND as an actual condition and public health concern when they participated in our study. Although there would likely be important differences in terms of cultural contexts and healthcare systems to consider, these findings are consistent with the results of previous research conducted by Gouse and colleagues that found healthcare providers in South Africa had already heard of HIV-related neurocognitive challenges or HAND, but lacked pertinent expertise in recognizing, addressing, and managing such challenges [10]. 

In our study, many providers reported that they engaged in different forms of continuing education opportunities, such as seminars, lunch-and-learns, and training, but noted that these opportunities rarely, if ever, covered topics related to the brain health of people aging with HIV/AIDS, and instead focused more on HIV medications or the clinical impacts of living with HIV/AIDS. It is important to consider that our participants expressed strong interest in gaining more learning opportunities in the future, particularly about the neurocognitive challenges that PLWH may experience, and that there is a clear path to disseminating critical information about HAND through increased continuing education and professional development focused on the neurocognitive aspects of living with HIV/AIDS. On a related matter, it is vital to note that our collaborative team’s decision to utilize a CBPR approach to not only investigate our research focus, but at the same time, to engage and create opportunities to educate relevant stakeholders throughout our research process (e.g., by advertising our study to stimulate interest during our recruitment phase, discussing HAND in greater detail during our participant interviews, disseminating a community report on our findings and lessons learned to providers and at their places of work after our thematic analysis) was a significant step towards successfully raising awareness and knowledge of HAND in the Southern Nevada HIV sector. Our decision to utilize a CBPR approach for our study was based on the documented merits of using it in studies dedicated to addressing health disparities that impact marginalized communities and to examining crucial issues related to the prevention, care, and treatment of middle-aged and older PLWH, such as organizational representation, partnership synergy, co-learning, community collaboration and empowerment, and checks on the dynamics of power and privilege [31,32,33].

In their interviews, our participants recalled instances at work where they encountered patients and clients who exhibited HAND signs and symptoms. They described older PLWH they worked with who grew increasingly forgetful or progressively required more assistance handling paperwork due to their neurocognitive decline. These work experiences are consistent with the findings of prior studies that have reported that PLWH experiencing neurocognitive challenges attributable to HAND struggle with their self-efficacy, become more dependent on others even with simple daily activities, and in particular, waver when navigating online (and other) health resources to meet their needs [16,37,38]. Our participants emphasized that lack of awareness and knowledge of HAND was a considerable barrier to supporting patients and clients. These findings stressed the need to disseminate information on HAND to providers who can further spread this information to their patients and clients, while also increasing their preparedness to address neurocognitive signs and symptoms of PLWH. It was apparent that our participants were already attending to these concerns and needs without necessarily realizing that they were dealing with issues that developed from their patients’ and clients’ HIV-related neurocognitive challenges. 

Based on our data, we were able to confirm that provider awareness is important for early detection of neurocognitive challenges that would allow PLWH to adopt valuable coping strategies and access relevant resources, as well as for providers to improve their HIV care with memory and other cognition-related services [39,40]. Several studies have already documented the inherent value of the early detection of the neurocognitive challenges of PLWH, which could then lead to the initiation or modification of patients’ or clients’ cART, and, potentially, the prevention of their functional decline and preservation or optimization of their physical functions [13,41,42,43]. For patients and clients experiencing neurocognitive challenges with lower levels of self-efficacy to advocate for themselves when interacting with providers [37], or for those struggling to navigate online and other health resources [38], provider awareness becomes a critical aspect of HIV care, as it enhances the ability of providers to ask important questions or to raise concerns that patients and clients aging with HIV/AIDS may not think of or remember to bring up. This is especially the case as we recognize that neurocognitive signs and symptoms need to be documented by or reported to a provider first before they can be addressed and managed in a treatment plan [44].

Healthcare and service providers in Southern Nevada have been providing a wide variety of programs and services in addition to basic HIV care in order to meet their patients’ and clients’ needs. Whether through their own programs and services or through their referrals to community partners, providers have connected PLWH to healthcare, food and housing assistance, transportation options, and job opportunities, to name a few. This finding is important because it not only underscores the fact that PLWH have a wide variety of needs that are created or exacerbated by their neurocognitive challenges, but it also underscores the point that providers are critical to keeping their patients and clients engaged in the HIV-care continuum, as well as connecting them to programs and services that help ensure their food, housing, and transportation security, which have been documented as critical social determinants of health that influence health disparities [42,45,46]. Our participants have been flexible and versatile with their job responsibilities, and have remained highly aware of vital resources available in the local community so that they can go above and beyond their duties to support PLWH. Beyond facilitating access to diagnostic and treatment services, many of our participants from the HIV sector have provided indispensable resources to PLWH in the community. They have helped address the adverse impacts of the neurocognitive decline of PLWH on known social determinants of health such as food and housing security, which could not only present obstacles in everyday life, but also jeopardize HIV treatment adherence [47,48,49]. They have also helped facilitate their patients’ and clients’ access to transportation options (e.g., through bus passes, and complimentary ride shares), which are essential for aging PLWH to get to various health-related appointments and much-needed social services [50,51]. 

Providers are critical to sustaining the HIV-care continuum. Their attitudes, connections with patients and clients, and established trust in communities engender effective engagement, treatment linkage, and retention [52,53,54]. Positive patient– provider relationships, and the availability of case managers and linkage-to-care coordinators, are essential to establishing comprehensive primary care for PLWH, further highlighting the importance of research examining provider perspectives and work experiences [55]. Previous research has explored and addressed provider perspectives on HIV care [56], but little work has been done to assess the awareness and knowledge of HAND among various providers in the 21st century [6,7,10]. 

Our study findings demonstrated a few key points. Firstly, there is room for improvement in terms of increasing awareness and knowledge of HAND, and many providers are open and eager to gain opportunities to learn more. Secondly, providers do notice HAND signs and symptoms in their patients and clients living with HIV/AIDS, whether they feel confident about their knowledge of HAND or not. This is a finding that could be utilized to its fullest potential through the promotion of appropriate HAND-focused continuing education and professional development opportunities. Thirdly, providers take on more responsibilities than merely providing basic HIV care, often facilitating access for PLWH to resources in their place of work or through referrals to community partners. It is paramount to recognize the crucial roles providers could play in terms of the early detection of the neurocognitive challenges of PLWH, and in providing resources that could address the adverse impact of these challenges on the lives of their patients and clients. Lastly, providers largely depend on a referral network that includes other providers, clinics, community health centers, agencies, and organizations to establish and sustain full and comprehensive care, programs, and services for people aging with HIV/AIDS and experiencing neurocognitive challenges. Future research could investigate new ways to bolster and enhance already-established HIV sector networks and referral systems of care. 

### Strengths and Limitations of the Study

Our study adds novel findings and important lessons learned to the limited current body of knowledge and academic literature on HIV sector healthcare and service providers’ awareness and knowledge of HAND, and additionally, contributes new insights on the prospective roles providers could play in the future to support aging PLWH experiencing neurocognitive challenges. However, it is important to acknowledge our study’s limitations. Our use of a purposive sampling technique [34] to recruit specific HIV sector healthcare and service providers from the community has excluded the examination of the awareness and knowledge of HAND of other providers (i.e., primary care providers with a clinical practice focused on HIV/AIDS, psychiatrists, healthcare providers in occupational treatment settings, neuropsychologists, and HIV specialists and researchers), and consequently, restricted the findings and lessons learned we gained. Furthermore, a strong interest and eager willingness to participate in our research focused on HAND, perhaps to learn more about the neurocognitive challenges of PLWH, may have led or prompted keen providers from our community to join our study, which in turn, may have influenced the types of responses we obtained in our interviews. Researcher bias and subjectivity could also have affected our research team’s qualitative thematic analysis. We utilized consensus coding among eight researchers to minimize this concern, but the perspectives of our research team are still those of university-based psychology scholars. Greater diversity among coders could prove useful in future related studies to avoid the possibility of overlooking themes that are typically not captured by the coding practices and mindset of many academic researchers.

## 5. Conclusions

In pursuit of its primary research aims, this CBPR study examined the awareness and knowledge of HAND among healthcare and service providers in the HIV sector of Southern Nevada, and additionally, it explored their prospective future roles in supporting aging people with HIV/AIDS experiencing neurocognitive challenges. Our findings revealed that, while providers demonstrated a general awareness of brain health impacts related to HIV/AIDS, specific knowledge of HAND could still be improved, and many providers are open and willing to learn more. Lack of awareness and knowledge of HAND was a key barrier to supporting PLWH with neurocognitive deficits. Our findings also revealed that continuing education opportunities to learn about HAND would improve providers’ abilities to identify and meet the needs of PLWH exhibiting neurocognitive challenges attributable to HAND. Furthermore, our study highlighted the critical roles providers already play in connecting PLWH to the wide range of services beyond basic HIV care, such as housing assistance, access to nutritious food, transportation options, and job opportunities, which are necessary to providing the full range of care for PLWH experiencing neurocognitive challenges. Future studies on this research focus could potentially explore different ways providers could sustain reliable opportunities to increase their awareness and knowledge of HAND, as well as investigating other crucial roles providers could play to support PLWH experiencing neurocognitive challenges.

Overall, our study contributes important and valuable insights that fill specific knowledge gaps in the current literature, and could help improve the capacity and skill sets of HIV sector healthcare and service providers in Southern Nevada. Our findings call for continued efforts from researchers, scholars, and relevant communities to improve awareness and knowledge of HAND, promulgate the critical roles providers could play in supporting PLWH with neurocognitive challenges, and augment access to relevant resources for PLWH and their providers. By heeding this call, HIV sector healthcare and service providers could facilitate the early detection of neurocognitive challenges among people aging with HIV/AIDS; provide much needed programs, services, and appropriate referrals; and institute comprehensive care and support for their patients and clients experiencing neurocognitive challenges.

## Figures and Tables

**Table 1:** Interview Guide Questions.

Could you please tell me about your practice/work as a healthcare or service provider in the HIV sector of Southern Nevada? Have you ever encountered any clients/patients living with HIV/AIDS in your work who may be experiencing changes in their neurocognitive functions or having brain health issues that may be related to HAND? Please elaborate. Why do you think some of your clients/patients have been experiencing these challenges or brain health issues? How long has it been since you have been aware of the existence of HAND as a condition that affects PLWH? Do you believe there are effective ways you and other providers could help address brain health issues related to HIV/AIDS or HAND? Please elaborate. How have you discussed brain health challenges related to HIV/AIDS with your clients/patients? What kind of strategies do you use, if any, to support your patients/clients living with HIV experiencing neurocognitive challenges?

## Data Availability

Data is unavailable due to privacy and ethical restrictions.
